# Pearl Kendrick, Grace Eldering, and the Pertussis Vaccine

**DOI:** 10.3201/eid1608.100288

**Published:** 2010-08

**Authors:** Carolyn G. Shapiro-Shapin

**Affiliations:** Grand Valley State University, Allendale, Michigan, USA

**Keywords:** Pearl Kendrick, Grace Eldering, pertussis, state departments of health, vaccines, whooping cough, bacteria, historical review

## Abstract

In light of the reemergence of pertussis (whooping cough), the pioneering research of Pearl Kendrick and Grace Eldering is worth revisiting. In the 1930s, working in the Michigan Department of Health laboratory in Grand Rapids, Michigan, USA, they began researching a pertussis vaccine. Their research offers an instructive case study of the creative public health research performed in state health department laboratories during the interwar years. State department of health laboratory directors actively promoted research by supporting advanced education; making facilities and funding available for individual projects; and, when possible, procuring new facilities. Using Michigan Department of Health resources and local and federal funding, Kendrick and Eldering developed standardized diagnostic tools; modified and improved extant vaccines; conducted the first successful, large-scale, controlled clinical trial of pertussis vaccine; and participated in international efforts to standardize and disseminate the vaccine. Their model may again offer a promising avenue for groundbreaking research.

## Abstract 2

State health department laboratories are crucial to the development of public health research.

In light of the re-emergence of pertussis (whooping cough), the pioneering pertussis vaccine research conducted by Drs Pearl Kendrick and Grace Eldering (Figure) at the Michigan Department of Health laboratory is worth revisiting. Their pertussis research offers a model that would be useful today.

**Figure:**
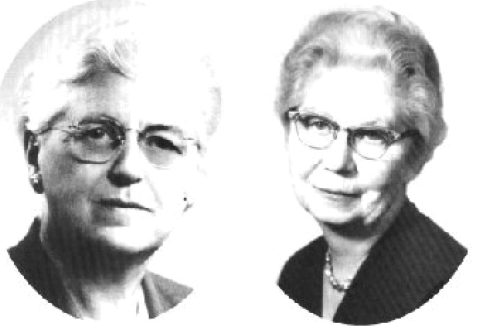
Pearl Kendrick (left) and Grace Eldering. Photo credit: Michigan Women’s Hall of Fame (www.michiganwomenshalloffame.org).

Although scientists had developed vaccines to control many infectious diseases including smallpox, typhoid fever, diphtheria, and tetanus by the 1920s, whooping cough proved a more difficult puzzle. French researchers Bordet and Gengou described *Bordetella pertussis* as the causative agent of whooping cough in 1906 (*1*). In the 1920s, pharmaceutical companies in the United States offered many pertussis and mixed-serum pertussis vaccines designed to both treat and prevent whooping cough, but none proved effective (*2*). In 1931, the American Medical Association's Council on Pharmacy and Chemistry found no “evidence even for the presumptive value of stock or commercial vaccines” because “the pertussis vaccines seem to have absolutely no influence [as a preventive], and after the disease is thoroughly established even freshly prepared vaccines seem useless” (*3*).

By the 1920s, pertussis had claimed the lives of ≈6,000 US children each year, more than did each of the childhood scourges of diphtheria, scarlet fever, and measles (*4*). Thorvald Madsen of the Danish Serum Institute in Copenhagen spurred further pertussis research when he announced that his vaccine prepared from freshly isolated *B. pertussis* cultures offered some protection in his Faroe Islands studies in the 1920s (*5*). English scientists P. H. Leslie and A. D. Gardner described 4 antigenic groups or phases for *B. pertussis* and highlighted the importance of selecting appropriate cultures for vaccine production in 1931 (*6*). Illinois pediatrician Louis Sauer and his assistant Leonora Hambrecht conducted smaller scale tests of their effective vaccine (*4*). Still, the disease remained a killer (*7*). In 1932, when Kendrick and Eldering began their research at the Michigan Department of Health laboratory in Grand Rapids, Michigan, USA, many questions remained unanswered.

Starting in the mid-19th century, public health leaders across the nation developed city and state departments of health. When bacteriology was introduced in the late 19th century, these health departments gradually shifted their mission from promoting general sanitation to public health efforts that targeted the specific vectors of disease and focused on laboratory diagnosis (*8**–**10*). By 1915, most major cities and all states had invested in laboratory facilities dedicated to bacteriologic analysis, biologics production, and (in many) fundamental research (*10**,**11*). After World War I, state health department laboratory directors expanded their laboratory divisions with funds newly available from the federal government, the American Public Health Association (APHA), and the Rockefeller Foundation (*9**,**10*). For Charles Chapin, Superintendent of Health for Providence, Rhode Island, however, most of these laboratories had not reached their potential because of limited funds and personnel. “On the whole,” he noted, “investigation of the sources of diseases has not attained very brilliant results in the hands of most state health departments, as their energies have been largely forced into other channels whether they wished it or not” (*10*).

To staff their growing departments and stay within their limited budgets, laboratory directors often sought out talented female scientists. Pearl Kendrick, from her days as a student and teacher in upstate New York, was recognized by her teachers as being “a first class student, thorough, accurate and rapid” (*12*). While teaching, she continued her own education, studying bacteriology at Columbia University under Hans Zinsser during the summer of 1917 (*12*). After Kendrick had worked for 2 years as an assistant at the New York State Department of Health laboratories, C.C. (Cy) Young, director of the Bureau of Laboratories for the Michigan Department of Health, recruited her. Young assured Kendrick that “I’m sure that we can make it interesting for you and there is every chance for advancement” (*12*). In 1926, Young assigned Kendrick to direct the health department’s newly opened Grand Rapids branch. Young provided his employees funding and time to pursue advanced education. In 1932, Kendrick earned a Doctor of Science degree in bacteriology from Johns Hopkins University. Young’s strategy of pursuing talent, supporting advanced education, and funding research paid dividends. By the late 1920s, Young’s Bureau of Laboratories in Lansing and Grand Rapids had established a national reputation for bacteriologic research.

Grace Eldering, Kendrick’s laboratory partner, hailed from eastern Montana. Eldering studied at the University of Montana in Missoula and taught English and biology at Hysham High School for a year after graduation before answering the Michigan Bureau of Laboratories’ call for additional staff. In the fall of 1928, Eldering traveled to Lansing to take advantage of this opportunity. Within 6 months, she was hired into the department to do routine bacteriologic analysis. Eldering would earn her Doctor of Science degree from Johns Hopkins University in 1942 (*13*).

In 1932, Kendrick brought Eldering to Grand Rapids, and, when a virulent strain of *B. pertussis* infected the children of Grand Rapids that year, Kendrick and Eldering began the whooping cough research project. The state laboratory gave Kendrick and Eldering the freedom to conduct their research after all of the laboratory’s routine water and milk analyses were completed. They developed and improved methods for growing the pertussis bacillus, inactivating it, and creating a safe vaccine (*14**,**15*). In addition, they were pioneers in the field, designing and directing the first large-scale controlled clinical trial for pertussis vaccine. In the pages of Reader’s Digest, Paul DeKruif, a Grand Rapids native and author of the best seller Microbe Hunters, celebrated their study as one of the “greatest field tests in microbe-hunting history” (*16*).

To pursue their groundbreaking research, Kendrick and Eldering brought together a diverse coalition of local and state public health departments, physicians, citizens’ groups, women’s groups, and parent–teacher associations that would provide organizational support and funding. By building relationships with local physicians and the Grand Rapids Health Department, Kendrick and Eldering ensured a steady supply of cough plates containing *B. pertussis* samples. When city physician A.H. Edwards notified them of pertussis infections in the community, Kendrick and Eldering sped off to visit families hit hard by the economic downturn and to collect samples. As Grace Eldering noted in an interview, they “learned about pertussis and the Depression at the same time” (*15*). In addition, the city health department aided their research by allowing its network of public and private nurses to collect cough plates for the pertussis research.

During the first stage of their research, Kendrick and Eldering modified the then-standard Bordet-Gengou growth medium; the result was a growth medium that fostered more rapid and profuse growth of *B. pertussis* colonies and that could therefore be used as a routine diagnostic tool (*15*). On November 1, 1932, Kendrick’s laboratory began offering a cough plate diagnostic service to local doctors. In addition to aiding the doctors, the rapid-growth plates enabled Kendrick and Eldering to determine that during the first 3 weeks of infection, a child’s cough contained enough active pertussis bacilli to infect his or her peers; that most children were noninfectious by week 4; and that after 5 weeks, 90% of the children posed no risk to others (*17*).

Proper quarantine length could now be determined scientifically. Before these studies, whooping cough quarantines varied from 2 to 4 weeks, depending on locale. The Grand Rapids Health Department adopted Kendrick and Eldering’s quarantine recommendations, which required physicians to report the disease; ordered the health department to place warning placards on homes; and enforced a 35-day isolation period or, with 2 consecutive negative cough plates, release by day 28 after onset of symptoms (*18*). As Pearl Kendrick noted in 1934, these “regulations have crystallized out of our bacteriological studies and are now under test as part of the Grand Rapids Communicable Disease Regulations” (*19*). In 1935, Kendrick reported that the “cough plate technic [sic] has become a routine procedure in the laboratory”; that year, Kendrick’s Michigan Department of Health laboratory in Grand Rapids examined 4,515 cough plates for *B. pertussis* (*20*).

After several months of producing autogenous vaccines for local physicians to use as treatment and preventive, Kendrick appealed to state laboratories director Cy Young for permission to develop a more general vaccine. “Rather than handl[ing] each request on the basis of an autogenous vaccine,” Kendrick explained to Young on February 4, 1933, “we can more efficiently make a supply from several local pertussis strains.” She then asked: “May we do this on an experimental basis—supplying these few pediatricians who are the type to cooperate as to records [?]” (*12*). Young supported her efforts. In a handwritten note dated February 21, 1933, he told Kendrick: “Go ahead and do all you can with pertussis if it amuses you” (*12*).

Kendrick and Eldering performed carefully controlled animal studies of vaccines, using the general methods of Madsen, Sauer, and Hambrecht to design a vaccine that was safer and more potent (*15*). They inactivated the pertussis bacilli with thimerosol at cold room temperature for >1 week and conducted numerous sterility and safety tests (including injecting the vaccine into their own arms to test for safety) (*12**,**14**,**15*). After these vaccines were declared safe, they were distributed to local physicians who, in return for the serum, supported Kendrick and Eldering’s laboratory work by spreading the news of the vaccine to area medical personnel and encouraging wider use of diagnostic cough plates (*12*).

Recruiting study participants and gathering financial support for a wide-scale vaccine trial required concerted community outreach efforts (*18**,**20*). In a 1958 retrospective on their field studies, Grace Eldering noted that “among the many who contributed to the success of the program were the parents and their children who accepted the requirements for test and control groups in the field trials. This acceptance was basic, and laid a foundation in the community upon which other studies could be built” (*21*).

In the 1930s, there were no accepted standards and few established models for conducting field studies, a problem made clear in failed experiments with human participants, including the Brodie-Park and Kolmer polio trials in the 1930s (*22*). Many researchers used orphans or institutionalized children for their research, noting that by participating, these children were repaying a debt to society (*23*). Instead of relying on these vulnerable populations, Kendrick and Eldering built outreach networks during the early stages of their research. The Kent County Welfare Relief Commission aided these efforts by collecting statistics on the prevalence of whooping cough and the number of children who had received “a treatment to prevent whooping cough” in their 1935 vaccination survey. This study of vaccination of preschool-aged children and the careful records of the city health department's nursing districts enabled Kendrick and Eldering to select controls matched for age, sex, and district (*12*).

Private physicians joined school physicians and city health officials in administering the series of 4 or 5 shots at the vaccination clinics held in primary schools around the city, federally funded nursery schools, and at City Hall (*12**,**24*). For each child in the study, city health department nurses completed a vaccine inoculation form, a home visit slip, an exposure record, and a case history; the researchers matched each inoculated child with a control selected from the Kent County Welfare Relief Commission study. At 3–4-month intervals, the nurses visited children in both groups and collected information about exposures; checked patients for the bacillus with cough plates; and when needed, obtained case histories for exposures and illness (*14*). The 1934–1935 field trial involved 1,592 (712 vaccinated and 880 control) children. In their 1935 report to the APHA, Kendrick noted that only 4 of the 712 vaccinated children had whooping cough, and then only mild cases, but 45 of the 880 unvaccinated controls (90% of those exposed) contracted the disease and suffered its full ravages. Despite the 89% efficacy rate found in the trial, they cautioned against the “danger of giving [the numbers] too much weight in the face of the relatively small number of whooping cough cases” (*14*).

Before the large-scale federal financing of science that emerged after World War II, scientists were often forced to cobble together funds from private and public sources (*25*). Kendrick and Eldering conducted their research on a shoestring budget. The Michigan Department of Health allowed them to use laboratory facilities after hours for their early pertussis research, and for the first 2 years of their studies, a total of $1,250 arrived from private citizens, the Grand Rapids City Commission, and the National Research Council. Later, and only after their vaccine showed progress, did they receive additional funding for staffing and research from the Federal Emergency Relief Administration (a New Deal Agency) and from the Michigan Department of Health (*15*). When, in early 1936, the whooping cough vaccine project’s funds again ran low, Kendrick invited Eleanor Roosevelt to visit their laboratory. Roosevelt helped secure the funds needed to add several Works Progress Administration workers to Kendrick and Eldering’s staff. In 1938, the Works Progress Administration furnished additional clerical staff, and the APHA helped defray the cost of statistical analysis (*26*). Funding from all sources for the study amounted to $181,695.60 (*12*). Later, the National Institutes of Health would fund additional pertussis studies, and the Michigan Department of Health would continue funding public health research into the 1980s.

That Kendrick and Eldering crafted a well-controlled trial is revealed in their successful defense of their research against the skepticism of public health leaders. Soon after they announced their vaccine results, James Doull, a prominent Cleveland epidemiologist, reported that children received no protection from his whooping cough vaccine. The APHA subcommittee on whooping cough, which included both Kendrick and Doull, evaluated the divergent results of the 2 studies. Unable to explain the difference in results, committee members then enlisted Wade Hampton Frost, a Johns Hopkins University epidemiologist and head of the APHA, to review the work. Although predisposed to find fault with Kendrick’s work because of his belief that few studies could meet strict standards of control (*12*), Frost journeyed twice to Michigan to inspect Kendrick’s findings and, in the end, supported her work. Frost noted to Kendrick, “I think it may be assumed, not as a conclusion but merely as a working hypothesis, that your data when finally analyzed are likely to show some protection in the vaccinated group. Therefore, without accepting this as a conclusion, I think it is proper to make plans for further work based on this presumption, and I would suggest two additional projects” (*12*). Had Frost not died shortly thereafter, this productive collaboration might have continued (*27**,**28*).

Encouraged by the results of the 1936 trials, parents in Grand Rapids flocked to enroll their children in Kendrick and Eldering’s 1938 follow-up study in which children were given smaller doses of the pertussis vaccine, administered in 3 injections. This new regimen was found to be as effective as the 4 injections given in the original study (*29*). On the basis of this study, the Michigan Department of Health Biologic Products Division began mass-producing the pertussis vaccine for children in Michigan in 1938, and, by 1940, pertussis vaccine was widely distributed across the nation.

In 1943, the American Academy of Pediatrics approved the vaccine for routine use; a year later, the American Medical Association recommended its use (*30*). The nation’s whooping cough incidence and death rates would drop dramatically. In 1934, the whooping cough incidence in the United States was 209 cases/100,000 residents, and the death rate was 5.9/100,000. By 1948, routine use of the vaccine reduced the incidence to 51 cases/100,000 residents and the death rate to <1/100,000. After 1960, incidence was <10 cases/100,000 residents (*31*).

In the early 1940s, Kendrick’s Michigan Department of Health laboratory participated actively in APHA Pertussis Study Group studies designed to standardize the pertussis vaccine (*28*). At this juncture, the public health community used adherence to a manufacturing process as the standard measure of their vaccine’s safety and efficiency, despite the fact that methods of inactivating the bacillus and manufacturing the vaccine varied widely. Before advocating wider dissemination of the vaccine, the APHA Pertussis Study Group worked closely with Kendrick and pharmaceutical companies to develop measurable standards and verifiable tests that could be applied to the end product regardless of the manufacturing process used. As Kendrick noted on March 16, 1942, to W.A. Feirer of Sharpe and Dohme, “May I repeat that in relation to the work of your committee on standards, it seems to me that the problem of first importance is to attempt to reach some degree of uniformity in judging the concentration of the organisms in the product. This does not mean necessarily that the same method of standardization be used by all manufacturers. It does mean that it should be possible to check their labeled concentrations within an accepted range of variation, by a single method” (*13*). Using APHA and pharmaceutical company funding, Kendrick and Eldering developed an opacity standard by adjusting “the turbidity of a suspension of Pyrex glass particles to be equivalent to that of a specified number of bacteria of an aged vaccine determined by direct count” (*32**,**33*). In 1946, the United States adopted this standard; in 1958, the World Health Organization designated it as the international standard.

Although the American medical community readily adopted Kendrick and Eldering’s whooping cough vaccine, the editor of the British Medical Journal expressed more skepticism, arguing that none of the American studies used proper control groups and that their own trials had shown such vaccines to be ineffective (*34*). David Evans of the British Medical Research Council (MRC) and J.S. Wilson of the London School of Hygiene did not share the British Medical Journal’s concerns; indeed, they turned to Pearl Kendrick to assist with the MRC’s next series of studies. Kendrick not only supplied the British with American serum to compare with the British vaccines and assisted in designing their study but also, with MRC funding, tested the potency of the vaccines, by using a mouse protection assay developed in the Grand Rapids laboratory, before the vaccines were used in the MRC field trials (*13**,**35*). In the 1950s, the World Health Organization and the Whooping Cough Immunization Committee of the MRC funded Kendrick’s trip to England so that she could review the MRC’s pertussis vaccine field trials (*35*).

Over the course of their careers, Kendrick and Eldering published >60 articles in a wide variety of journals, including the American Journal of Public Health, the Journal of Infectious Diseases, the American Journal of Hygiene, the Journal of Bacteriology, the Journal of Pediatrics, and the Journal of Laboratory and Clinical Medicine; they received frequent requests for reprints (*13*). Kendrick’s thick correspondence files make clear that they shared their vaccines, plates, cultures, and research with scientists around the world and hosted many international visitors in their laboratory. Kendrick traveled the world, often as a consultant for the World Health Organization, helping to establish vaccine programs in Mexico, eastern Europe, and Central and South America. In 1962, she served as part of an exchange delegation on immunology to the Soviet Union (*13**,**35*).

Kendrick and Eldering participated actively in the inner circles of the international bacteriology and public health communities. Indeed, they were well known in scientific circles for their gracious hospitality at the dinner parties and picnics they hosted at their Grand Rapids home (*13*). Still, they did not seek the traditional rewards of fame, despite the many opportunities offered later in life. Indeed, they actively shunned publicity, turning down opportunities to appear on the Today Show as so much attention was, in the words of Grace Eldering, “embarrassing” (*36*).

Shortly after Kendrick’s death, Dean Richard Remington, writing in the University of Michigan’s School of Public Health newsletter, noted:

A life saved by prevention cannot even be identified. Who are the men and women living today who would be dead from whooping cough had it not been for Pearl Kendrick’s vaccine? We can conclude with reasonable certainty that several hundred thousand of them are now leading productive lives, in this country alone. But who are they? Name one. You can’t do it and neither can I.…The accomplishments of disease prevention are statistical and epidemiological. Where’s the news value, the human interest in that? … But a public service orientation can provide more than ample compensation. Dr. Kendrick never became rich and, outside a relatively small circle of informed friends and colleagues, never became famous. All she did was save hundreds of thousands of lives at modest cost. Secure knowledge of that fact is the very best reward (*37*).

In recent years, state department of health laboratories have lost personnel and much of their research funding (*38*). Kendrick and Eldering’s model of enlisting the support and resources of the local, state, and national communities may once again offer a promising avenue for conducting groundbreaking research.
